# Associations Between Loneliness Components and Nomophobia Among Allied Medical Sciences Students in Sari: A Cross‐Sectional Study

**DOI:** 10.1002/hsr2.73195

**Published:** 2026-09-03

**Authors:** Hasan Siamian, Azita Balaghafari, Forouzan Elyasi, Shahram Mollania‐Jelodar, Zahra Mahmoudvand, Azadeh Yazdanian

**Affiliations:** ^1^ Department of Health Information Technology, School of Allied Medical Sciences Mazandaran University of Medical Sciences Sari Iran; ^2^ Psychiatry and Behavioral Sciences Research Center, Addiction Institute Mazandaran University of Medical Sciences Sari Iran; ^3^ Department of Social Sciences Payam Noor University Tehran Iran

**Keywords:** allied medical sciences, loneliness, mental health, mobile phone use, nomophobia, university students

## Abstract

**Background and Aims:**

Nomophobia, the fear or anxiety of being without access to a mobile phone, has become increasingly common among university students due to the widespread integration of smartphones into daily life. Although previous studies have suggested a link between loneliness and nomophobia, evidence from Iranian Allied Medical Sciences Students remains limited. This study aimed to investigate the association between loneliness and the severity of nomophobia among students of the School of Allied Medical Sciences at Mazandaran University of Medical Sciences in Sari, Iran.

**Methods:**

This cross‐sectional analytical study was conducted among undergraduate students (*n* = 248) during the first semester of the 2024 academic year. Participants were selected using proportionate stratified random sampling. Data were collected using the UCLA Loneliness Scale (Version 3) and the Nomophobia Questionnaire (NMP‐Q). Descriptive statistics, independent samples *t*‐tests, chi‐square tests, Pearson's correlation coefficient, and simple linear regression were used for data analysis. Statistical significance was set at *p* < 0.05.

**Results:**

A total of 248 students participated in the study. Overall, participants reported moderate levels of nomophobia and low‐to‐moderate levels of loneliness. Pearson correlation analysis revealed a weak but statistically significant positive correlation between loneliness and nomophobia (*r* = 0.156, *p* = 0.014). Simple linear regression demonstrated that loneliness was a significant predictor of nomophobia, explaining 2.4% of the variance (*F*(1, 246) = 6.12, *p* = 0.014, *R* ^2^ = 0.024). Multiple linear regression analysis indicated that the overall model was statistically significant (*F*(13, 230) = 3.965, *p* < 0.001). Field of study (*p* = 0.005), marital status (*p* < 0.001), duration of smartphone use (*p* < 0.001), and frequency of smartphone checking (*p* = 0.021) emerged as significant predictors of nomophobia. After adjustment for these variables, loneliness remained an independent predictor of nomophobia (β = 0.149, *p* = 0.014).

**Conclusion:**

Loneliness was identified as a significant, albeit weak, predictor of nomophobia among students of Allied Medical Sciences. Although loneliness contributed to nomophobia, the findings indicate that smartphone‐related anxiety in this population is influenced by a broader combination of psychosocial, behavioral, and academic factors. Interventions aimed at reducing nomophobia should therefore adopt a multifaceted approach that promotes both students' psychological well‐being and healthy smartphone use behaviors.

## 1 Introduction

Loneliness refers to the personal and emotional experience of feeling socially isolated or disconnected, which differs from the actual, measurable absence of social interactions known as objective social isolation [1]. Loneliness is a complex psychological construct that has long attracted the attention of philosophers and psychologists throughout history. Although in the past loneliness was often regarded as a voluntary withdrawal from social relationships and an opportunity for personal growth, in contemporary psychology it is mostly recognized as a negative and unpleasant experience resulting from the absence or poor quality of interpersonal relationships, and is associated with consequences such as depression, anxiety, and social withdrawal [2, 3].

Loneliness has long been recognized as a universal phenomenon with significant effects on individuals' mental health [4]. It adversely affects well‐being and has long‐lasting negative consequences for physical and mental health, academic performance, and employability. Due to these ongoing and lasting effects, loneliness is a significant issue that requires reliable and appropriate measurement tools [5]. In today's era, often called the “Information and Communication Age,” technological advancements have dramatically transformed the ways people interact with each other [6]. Excessive engagement in compulsive internet use, gaming addiction, and social media use has been associated with higher levels of despair, loneliness, poor sleep quality, and anxiety among students [7]. Supporting the psychological relevance of loneliness in mobile‐related behaviors, Bai et al. (2025) showed through a cross‐lagged analysis that loneliness and shyness are significantly associated with mobile phone addiction among university students [8]. Furthermore, recent evidence suggests that loneliness may interact with other psychological characteristics in the context of problematic mobile use. Fang et al. (2025) showed that loneliness and self‐concept clarity play important roles in the relationship between problematic mobile social network usage and social anxiety among college students [9]. Consistent with this pattern, Wang et al. (2025) reported that loneliness was positively associated with mobile phone addiction among college students, with mobile phone anthropomorphism and interpersonal relationship quality acting as mediating and moderating factors [10]. The clinical and practical importance of loneliness among college students has also been highlighted by recent intervention research. Nguyen et al. (2025) demonstrated that a mobile‐based relational savoring intervention was feasible, acceptable, and showed preliminary benefits in preventing loneliness in this population [11].

Nomophobia, derived from “no‐mobile‐phone phobia,” encapsulates the fear and anxiety associated with the absence of smartphone or internet connectivity [12, 13, 14, 15]. It is characterized as a contemporary phobia that has emerged in the digital age, referring specifically to the anxiety experienced when an individual is separated from their mobile device. While commonly understood as a fear related to communication disruption via mobile phone or the internet, nomophobia is also viewed as a multidimensional condition encompassing social, physiological, and psychological symptoms [16, 17]. Given the widespread adoption of smartphones, its prevalence has significantly increased, placing high‐risk populations such as university students at greater susceptibility [13, 18, 19].

Students, as the age group at the forefront of adopting new communication technologies, are among the heaviest users of smartphones, which have become a central tool for their social interactions [20]. The extensive and frequent use of smartphones, especially among paramedical students, has led to consequences such as nomophobia, which has become a comparatively common psychological issue in this population [16, 18]. Hanley (2013) noted that the younger generation uses smartphones not only for texting but also for various activities such as emailing, listening to music, downloading content, and engaging with social networks, which have transformed their ways of communication [21].

A growing body of research has revealed multiple types of relationships between nomophobia and feelings of loneliness [22]. Although mobile phone addiction and nomophobia are conceptually distinct—addiction involving excessive use and nomophobia involving anxiety about disconnection—both have been linked to loneliness. For instance, a qualitative study reported a strong connection between mobile phone addiction and loneliness [23]. Recent studies also suggest that loneliness may contribute to nomophobia through emotion regulation difficulties and attachment‐related factors. Excessive smartphone use can lead to significant physical and psychological consequences; prominent symptoms of nomophobia include anxiety, changes in breathing, trembling, sweating, mental agitation, and increased heart rate [12, 24]. Based on the findings of Lepp et al. (2014), the average daily smartphone usage time for students is approximately 300 min [25], and Clayton et al. (2015) found that smartphone users send an average of 109 text messages per day [24]. Excessive smartphone use can therefore be considered a type of addictive behavior that reduces an individual's control over their phone usage [12].

Previous studies have shown that loneliness is directly associated with the severity of nomophobia and may, in some models, exert indirect effects through factors such as smartphone dependence, attachment anxiety, and emotion regulation difficulties. These mechanisms have been reported in prior literature and were not the focus of the present study [26, 27]. Yildirim and Correia (2015) identified the main dimensions of nomophobia as the inability to communicate, fear of losing connection, limited access to information, and loss of convenience [28]. More recent findings suggest that the impact of loneliness on nomophobia may operate through pathways involving emotion regulation and social anxiety [26].

Despite these findings, the association between loneliness and nomophobia among Iranian Allied Medical Sciences Students remains insufficiently explored. In particular, there is limited evidence regarding how loneliness is related to nomophobia among students in Iranian allied medical sciences schools, especially those studying in Sari.

The present study aimed to investigate the association between loneliness and nomophobia among undergraduate allied medical sciences students. These students represent an academically and clinically active population whose frequent use of smartphones for communication, scheduling, and educational purposes may place them at increased relevance for nomophobia research. In this study, demographic and academic characteristics (including age, sex, marital status, place of residence, field of study, and year of study), as well as smartphone‐use variables (duration of use and frequency of phone checking), were examined to provide a clearer understanding of potential correlates of nomophobia.

Accordingly, the guiding research question of the study was: Is loneliness associated with nomophobia, and to what extent does loneliness predict nomophobia after accounting for key demographic and smartphone‐use variables?

## 2 Materials and Methods

### 2.1 Study Design and Setting

This cross‐sectional analytical study was conducted among undergraduate students (*n* = 248) during the first semester of the 2024 academic year, Sari, Iran. The study population consisted of 726 students. Based on the Krejcie and Morgan table (95% confidence level, 5% margin of error), the required sample size was calculated as 248. A proportionate stratified random sampling method was applied according to academic discipline, including operating room technology, anesthesiology, radiology, laboratory sciences, health information technology, and occupational therapy. All selected students participated and were included in the final analysis (*n* = 248).

A structured questionnaire collected information on demographic and academic variables, including age, sex, marital status, residence, academic discipline, and smartphone use patterns.

### 2.2 Measures

A structured questionnaire collected information on demographic and academic variables, including age, sex, marital status, residence, academic discipline, and smartphone use patterns.

The variables assessed in this study included demographic characteristics (age, sex, marital status, place of residence), academic factors (field of study and year of study), and patterns of smartphone use (daily duration of smartphone use and frequency of phone checking). Psychological variables consisted of loneliness and nomophobia, the latter assessed across its four established dimensions: communication inability, loss of connectedness, limited access to information, and loss of convenience. These variables were included to both characterize the sample and examine their potential associations with nomophobia.

### 2.3 Loneliness

Loneliness was assessed using the UCLA Loneliness Scale (Version 3) developed by Russell (1996) [29]. The instrument consists of 20 items designed to measure subjective feelings of loneliness and perceived social isolation. Items are rated on a 4‐point Likert scale ranging from 1 (never) to 4 (often), with higher total scores indicating greater loneliness. In the present study, loneliness was treated as a unidimensional construct and analyzed using the total score. The UCLA Loneliness Scale demonstrated good internal consistency in this study (Cronbach's α = 0.846).

### 2.4 Nomophobia

Nomophobia was assessed using the 20‐item Nomophobia Questionnaire (NMP‐Q) developed by Yildirim and Correia (2015) [28]. The Persian version of the NMP‐Q, validated in Iran in 2018, reported a Cronbach's alpha coefficient of 0.92 for internal consistency [30].

It consists of 20 items rated on a 7‐point Likert scale ranging from 1 (strongly disagree) to 7 (strongly agree). The scale comprises four dimensions: not being able to access information (4 items), giving up convenience (5 items), not being able to communicate (6 items), and losing connectedness (5 items). Total scores range from 20 to 140, with higher scores indicating higher levels of nomophobia. In the present study, the scale demonstrated good internal consistency (Cronbach's α = 0.886).

### 2.5 Statistical Analysis

Statistical analyses were performed using SPSS software (Version 26). Descriptive statistics, including frequencies, percentages, means, and standard deviations, were used to summarize participants' demographic characteristics and the main study variables. The normality of continuous variables was assessed using the Shapiro–Wilk test, as well as skewness and kurtosis indices. As the main variables were approximately normally distributed, parametric tests were applied for inferential analyses.

Independent samples *t*‐tests were used to compare mean nomophobia scores across binary demographic variables, such as gender and marital status. One‐way analysis of variance (ANOVA) was conducted to examine differences in nomophobia scores across age groups and fields of study. Chi‐square tests were used to evaluate associations between categorical demographic variables and nomophobia severity levels. Pearson correlation analysis was performed to assess the linear relationship between loneliness and nomophobia. Multiple linear regression analysis was further conducted to identify significant predictors of nomophobia. To ensure the validity of the regression analyses, model assumptions were examined prior to interpretation; specifically, the normality of residuals, homoscedasticity, linearity, and multicollinearity were assessed using standard diagnostic procedures, and no violations were detected.

In addition to statistical significance testing, effect sizes were reported to indicate the magnitude of observed differences, including Cohen's d for *t*‐tests, eta squared for ANOVA, and Cramér's V for Chi‐square analyses. All statistical tests were two‐tailed, and a *p*‐value of less than 0.05 (*p *< 0.05) was considered statistically significant.

### 2.6 Ethical Considerations

The study was approved by the Ethics Committee of Mazandaran University of Medical Sciences (IR.MAZUMS.REC.1401.10531). Written informed consent was obtained from all participants prior to data collection. Participation was voluntary, and the confidentiality and anonymity of responses were ensured.

## 3 Results

### 3.1 Demographic Characteristics of the Participants

A total of 248 participants were included in the final analysis. The demographic characteristics and mobile phone use patterns of the participants are presented in Table 1.

**Table 1:** Demographic characteristics and mobile phone use patterns of the participants (*N* = 248).

Variable	Category	*N*	%
Gender	Female	196	79.0
	Male	52	21.0
Age group (years)	18–22	154	62.1
	≥ 23	94	37.9
Field of study	Laboratory sciences	39	15.7
	Radiology	51	20.6
	Anesthesia	29	11.7
	Operating room	56	22.6
	Occupational therapy	19	7.7
	Health information technology	54	21.8
University year	First year	169	68.1
	Second year	49	19.8
	Third year	30	12.1
Place of residence	Urban	174	70.2
	Rural	74	29.8
Marital status	Single	203	81.9
	Married	45	18.1
Mobile data usage	Yes	177	71.4
	No	71	28.6
Duration of smartphone use	< 1 year	115	46.4
	2– < 3 years	3	1.2
	3– < 4 years	7	2.8
	≥ 4 years	123	49.6
Frequency of checking phone	Every 5 min	37	14.9
	Every 10 min	33	13.3
	Every 20 min	77	31.0
	Every 30 min	47	19.0
	Every 1 h	19	7.7
	Every 2 h	16	6.5
	Every 3 h	9	3.6
	> 3 h	10	4.0

*Note:* Values are presented as number (*n*) and percentage (%).

Table 1 presents the demographic characteristics of the 248 participants. The majority of the sample were female (79.0%) and aged 18 to 22 years (62.1%). Most participants were first‐year university students (68.1%), residing in urban areas (70.2%), and single (81.9%). The most common fields of study were Operating Room (22.6%), Health Information Technology (21.8%), and Radiology (20.6%).

### 3.2 Reliability and Association Between Loneliness and Nomophobia

The internal consistency of the study instruments was evaluated using Cronbach's alpha. The UCLA Loneliness Scale demonstrated good internal reliability (α = 0.846), while the Nomophobia Questionnaire (NMP‐Q) showed excellent internal consistency (α = 0.886). The NMP‐Q assesses four dimensions of nomophobia: not being able to communicate, losing connectedness, not being able to access information, and giving up convenience.

Pearson's correlation analysis was performed to examine the association between loneliness and nomophobia. The results indicated a weak but statistically significant positive correlation between loneliness and nomophobia (*r* = 0.156, *p* = 0.014), suggesting that higher levels of loneliness were associated with greater nomophobia.

To further evaluate this relationship, a simple linear regression analysis was conducted with loneliness as the independent variable and nomophobia as the dependent variable. The regression model was statistically significant (*F*(1, 246) = 6.12, *p* = 0.014) and explained 2.4% of the variance in nomophobia (*R* ^2^ = 0.024). Loneliness emerged as a significant positive predictor of nomophobia (β = 0.149, *p* = 0.014).

Descriptive statistics and Pearson correlation coefficients for the primary study variables are presented in Table 2. Loneliness scores ranged from 28 to 67 (*M* = 46.72, SD = 8.15), whereas nomophobia scores ranged from 20 to 126 (*M* = 82.33, SD = 17.63). Consistent with the regression findings, Pearson's correlation analysis confirmed a weak but statistically significant positive association between loneliness and nomophobia (*r* = 0.156, *p* = 0.014). This finding indicates that students reporting higher levels of loneliness were more likely to experience higher levels of nomophobia.

**Table 2:** Descriptive statistics and percent of maximum possible (POMP) scores.

Variable	Possible range	Observed range	Mean	SD	Loneliness	Nomophobia
Loneliness	20–80	28–67	46.72	8.15	—	0.156*
Nomophobia	20–140	20–126	82.33	17.63	0.156*	—

*Note:* Correlations are Pearson coefficients.

Abbreviations: MMM = mean, SDSDSD = standard deviation.

* *p* < 0.05.

### 3.3 Dimensions of Nomophobia

Descriptive statistics were calculated for the four dimensions of the Nomophobia Questionnaire (NMP‐Q) to characterize the distribution and relative intensity of nomophobia‐related symptoms among the participants. Because the four dimensions comprise different numbers of items and therefore different theoretical score ranges, raw dimension scores are presented alongside standardized Percent of Maximum Possible (POMP) scores. Reporting POMP values facilitates direct comparison of symptom intensity across dimensions while preserving the original scoring structure of the NMP‐Q. Table 3 summarizes the number of items, theoretical and observed score ranges, mean scores, standard deviations, and POMP values for each dimension.

**Table 3:** Descriptive statistics for the four dimensions of the nomophobia questionnaire (NMP‐Q), including standardized percent of maximum possible (POMP) scores (*N* = 248).

Dimension	No. of items	Theoretical score range	Observed score range	Mean ± SD	POMP (%)
Not being able to access information	4	4–28	4–28	17.64 ± 4.69	58.50
Losing connectedness	5	5–35	5–32	19.24 ± 5.43	47.47
Not being able to communicate	6	6–42	6–42	26.64 ± 6.70	57.33
Giving up convenience	5	5–35	5–31	19.98 ± 5.25	49.93

*Note: N* = 248. Higher scores indicate higher levels of nomophobia. Percent of Maximum Possible (POMP) scores were calculated as: POMP = ((Observed Mean − Minimum Possible Score)/(Maximum Possible Score − Minimum Possible Score)) × 100.

As shown in Table 3, the Not being able to access information and Not being able to communicate dimensions exhibited the highest standardized POMP scores (58.50% and 57.33%, respectively), indicating comparatively higher levels of nomophobia in these domains. In contrast, the Losing connectedness (47.47%) and Giving up convenience (49.93%) dimensions showed relatively lower standardized scores. Overall, these findings suggest that concerns related to access to information and communication were the most prominent dimensions of nomophobia in the present study population.

### 3.4 Nomophobia Severity and Prevalence among Students

Nomophobia severity was assessed using the total scores of the Nomophobia Questionnaire (NMP‐Q) and classified according to the cutoff values proposed by Yildirim and Correia (2015) [28]. The distribution of participants across the four severity categories is presented in Table 4.

**Table 4:** Distribution of nomophobia severity levels based on total NMP‐Q scores (*N* = 248).

Nomophobia level	Score range	*N*	%
Absence	20	5	2.0
Mild	21–59	42	16.9
Moderate	60–99	148	59.7
Severe	≥ 100	53	21.4

*Note: N = *248. Nomophobia severity categories were defined according to the cutoff values proposed by Yildirim and Correia (2015).

As shown in Table 4, only 2.0% of participants were classified as having no nomophobia (NMP‐Q score = 20). Mild nomophobia was observed in 16.9% of participants, whereas the majority exhibited moderate nomophobia (59.7%). Furthermore, 21.4% of students were classified as having severe nomophobia.

Overall, 98.0% of participants exhibited at least some degree of nomophobia, highlighting the high prevalence of nomophobia among students of the Faculty of Allied Medical Sciences. Moreover, 81.1% of participants were classified as having moderate‐to‐severe nomophobia, indicating that higher levels of nomophobia were common in this population.

### 3.5 Loneliness Levels

To provide a clearer descriptive understanding of loneliness among the participants, the total score of the UCLA Loneliness Scale was categorized into three ordinal levels: low, moderate, and high loneliness. This categorization was performed to summarize the overall distribution of loneliness severity within the sample and to facilitate interpretation of the distribution of loneliness levels within the study population.

The frequency and percentage distribution of participants across these loneliness levels are presented in Table 5.

**Table 5:** Distribution of loneliness levels With 95% confidence intervals (*N* = 248).

Loneliness level	*N*	(%)	95% CI for %
Low	41	16.5	12.2–21.7
Moderate	133	53.6	47.3–59.8
High	74	29.8	24.2–35.9

*Note: N* = 248. Percentages may not sum to exactly 100% due to rounding. Confidence intervals were calculated using the Wilson method for binomial proportions.

As shown in Table 5, more than half of the participants were classified as having moderate loneliness (53.6%, 95% CI: 47.3–59.8), making it the most prevalent loneliness category in the sample. Nearly one‐third of participants were classified as having high loneliness (29.8%, 95% CI: 24.2–35.9), representing a substantial proportion of the study population with elevated loneliness scores. In contrast, a smaller proportion of participants were classified within the low loneliness category (16.5%, 95% CI: 12.2–21.7). Overall, the confidence intervals indicate relatively precise estimates of the observed proportions, suggesting that moderate and high levels of loneliness were common among participants.

### 3.6 Associations Between Loneliness Levels and Demographic Characteristics

To examine whether loneliness levels differed according to participants' demographic characteristics, Pearson's chi‐square tests were conducted. These analyses evaluated the association between categorized loneliness levels (low, moderate, and high) and selected demographic variables, including gender, age group, field of study, university year, place of living, and marital status. The results of these analyses are presented in Table 6.

**Table 6:** Associations between loneliness levels and demographic characteristics.

Demographic variable	*N*	χ^2^	df	*p* value	Cramér's V
Gender	248	0.72	2	.699	.054
Age group	248	8.39	12	.754	.092
Field of study	248	8.49	10	.582	.093
University year	248	0.76	4	.944	.055
Place of living	248	0.50	2	.780	.045
Marital status	248	4.75	8	.784	.089

*Note: N* = 248. Pearson's Chi‐square tests were used to examine associations between demographic characteristics and categorized loneliness levels. Cramér's V was calculated as a measure of effect size.

As shown in Table 6, none of the examined demographic characteristics were significantly associated with loneliness levels. All chi‐square tests were non‐significant (*p* > 0.05), indicating that the distribution of low, moderate, and high loneliness categories did not differ significantly across demographic groups.

The effect sizes, as indicated by Cramér's V, were small for all demographic variables (all *V* < 0.10), suggesting negligible associations between demographic characteristics and loneliness levels. These findings indicate that loneliness categories were distributed relatively consistently across participants regardless of demographic characteristics.

Overall, the results suggest that demographic characteristics, including gender, age group, field of study, university year, place of living, and marital status, were not significant determinants of loneliness levels in this sample.

### 3.7 Differences in Nomophobia Scores Across Demographic Characteristics

To examine whether nomophobia scores differed according to participants' demographic characteristics, independent‐samples *t* tests were performed for variables with two categories (gender, dichotomized age group, and marital status), whereas one‐way analysis of variance (ANOVA) was conducted for variables with more than two categories (field of study). The results are presented in Table 7.

**Table 7:** Differences in nomophobia scores according to demographic characteristics among allied medical sciences students.

Variable	Category (*n*)	Mean ± SD	Test	Statistic	df	*p* value	Effect size
Gender	Female (196)	82.56 ± 19.02	Independent‐samples *t* test	t = 0.55	140.19	.585	Cohen's d = 0.07
	Male (52)	81.44 ± 11.04					
Age group (dichotomized)	18–22 (160)	80.1 ± 22.3	Independent‐samples *t* test	t = 2.18	246	.031*	Cohen's d = 0.36
	≥ 23 (88)	72.5 ± 18.9					
Marital status	Single (210)	79.0 ± 21.4	Independent‐samples *t* test	t = 1.72	246	.087	Cohen's d = 0.29
	Married (38)	73.2 ± 18.7					
Field of study	Six academic programs	—	One‐way ANOVA	F(5,242) = 1.01	5,242	.442	η^2^ = 0.02

*Note: p* < 0.05 indicates statistical significance.

As shown in Table 7, nomophobia scores did not differ significantly according to gender, marital status, or field of study. However, when age was analyzed as a dichotomized variable (18–22 vs. ≥ 23 years), younger students reported significantly higher nomophobia scores than older students (*t* = 2.18, *p* = 0.031, Cohen's *d* = 0.36), indicating a small‐to‐moderate effect size.

### 3.8 Differences in Nomophobia Scores across the Original Age Groups

Because age was initially categorized into four groups, an additional one‐way ANOVA was conducted to determine whether nomophobia scores differed across the original age‐group classification (18–22, 23–27, 28–32, and > 33 years). Prior to conducting the analysis, the assumptions of normality and homogeneity of variances were assessed. The results are presented in Table 8.

**Table 8:** One‐Way ANOVA comparing nomophobia scores across the original age groups.

Source of variation	Sum of squares	df	Mean square	F	*p* value
Between groups	1031.605	3	343.868	1.063	.366
Within groups	78952.378	244	323.575		
Total	79983.984	247			

*Note:* Effect size: η^2^ = 0.013 (small).

As shown in Table 8, the one‐way ANOVA revealed no statistically significant differences in nomophobia scores across the four age groups, *F*(3, 244) = 1.063, *p* = 0.366, with a small effect size (η^2^ = 0.013). Because Levene's test indicated unequal variances (*p* = 0.012), Games–Howell post hoc comparisons were performed. Neither the Games–Howell nor Tukey HSD procedures identified statistically significant pairwise differences between age groups (all *p* > 0.05).

The difference between the dichotomized age‐group analysis (Table 7) and the four‐category age‐group analysis (Table 8) may be attributed to the different approaches used for categorizing age. While the independent‐samples *t* test compared two broader age groups (18–22 vs. ≥ 23 years), the one‐way ANOVA examined differences across the original four age categories. Overall, age‐group differences were not statistically significant when age was analyzed using its original classification.

## 4 Discussion

The present study examined the relationship between loneliness and nomophobia among students of Allied Medical Sciences. The findings revealed a weak but statistically significant positive association between loneliness and nomophobia (*r* = 0.156, *p* = 0.014). Simple linear regression further demonstrated that loneliness was a significant positive predictor of nomophobia (β = 0.149, *p* = 0.014), accounting for approximately 2.4% of the variance. Although these findings indicate that loneliness contributes to nomophobia, the small effect size suggests that it represents only one of several factors underlying smartphone‐related anxiety in this population.

The observed association is consistent with previous research linking loneliness to problematic smartphone use and mobile phone addiction. Bai et al. (2025), for example, reported reciprocal relationships among loneliness, shyness, and mobile phone addiction in university students. Similarly, previous studies have suggested that individuals experiencing greater loneliness may rely more heavily on smartphones to compensate for unmet social needs, thereby increasing their vulnerability to nomophobia [8]. In the present study, participants reported moderate levels of nomophobia and low‐to‐moderate levels of loneliness, indicating that even relatively modest increases in loneliness may be associated with greater dependence on smartphone availability.

Most participants were women aged 18–22 years, reflecting the demographic profile of many Allied Medical Sciences programs. Although demographic variables were primarily included to characterize the study population, previous research has suggested that younger adults and women may be more susceptible to nomophobia [18, 31]. However, evidence regarding the influence of age and gender remains inconsistent. Moreno‐Guerrero et al. (2021) found that demographic characteristics were not consistently associated with nomophobia across different populations, suggesting that cultural context, educational environment, and measurement approaches may partly explain these discrepancies [32]. Likewise, recent meta‐analytic evidence has demonstrated substantial heterogeneity in the reported prevalence of smartphone addiction, emphasizing the influence of methodological and contextual differences across studies [33].

The UCLA Loneliness Scale and the Nomophobia Questionnaire demonstrated good internal consistency in the present study, supporting their reliability in this population. These findings are consistent with previous psychometric evaluations of both instruments among university students [34, 35]. Nevertheless, some researchers have emphasized the importance of establishing cultural validity before interpreting specific dimensions of loneliness or nomophobia [12]. Future studies employing confirmatory factor analysis across diverse populations may further strengthen the evidence for their structural validity.

Although loneliness was significantly associated with nomophobia, the relationship was relatively weak. One possible explanation is the functional role of smartphones in health sciences education. Students frequently depend on smartphones for academic communication, online learning, clinical coordination, and immediate access to educational and medical resources. Consequently, concerns about losing access to information or communication may arise primarily from academic and professional demands rather than emotional loneliness alone. Moreover, psychosocial factors such as perceived social support, resilience, and coping strategies may buffer the impact of loneliness on smartphone‐related anxiety. Previous studies have shown that perceived social support can mitigate the psychological consequences of loneliness and reduce vulnerability to problematic technology use [28, 36, 37].

From a theoretical perspective, the present findings can also be interpreted through the Compensatory Internet Use Theory (CIUT), which proposes that individuals may use digital technologies to compensate for unmet psychological or social needs. Although loneliness was associated with nomophobia in the present study, the relatively small effect size suggests that smartphone dependence among Allied Medical Sciences students may not be driven solely by social compensation. Instead, smartphones likely serve multiple concurrent functions, including educational activities, clinical communication, and access to academic resources. Similarly, emotion regulation models suggest that smartphones may be used to manage stress, uncertainty, or negative affect rather than loneliness alone. Within academically demanding health sciences programs, students may therefore develop smartphone dependence as a strategy for regulating academic‐related stress and maintaining perceived competence and connectedness. This interpretation is also consistent with contextual academic dependency frameworks, which emphasize that smartphones have become integral tools for learning, clinical coordination, and professional communication. Consequently, anxiety arising from smartphone unavailability may reflect disruption of essential academic functions in addition to interpersonal disconnection.

Despite the relatively small contribution of loneliness, nomophobia levels were generally moderate in this cohort. This finding is consistent with previous studies among Iranian health sciences students. Aslani et al. (2025) reported that approximately 72% of nursing students experienced moderate to severe nomophobia, with educational level, academic performance, and frequency of smartphone checking emerging as significant predictors [38].

Overall, the findings suggest that nomophobia among Allied Medical Sciences students is best understood as a multifactorial phenomenon shaped by emotional vulnerability, behavioral habits, and contextual academic demands. Accordingly, loneliness should be viewed as one contributing factor within a broader network of psychological and environmental determinants rather than as the primary driver of nomophobia.

### 4.1 Strengths and Limitations

This study has several notable strengths. It examined the relationship between loneliness and nomophobia in students of Allied Medical Sciences, a population that remains underrepresented in the literature. Validated instruments with good psychometric properties were used to assess both constructs, and proportionate stratified random sampling enhanced the representativeness of the study sample.

Several limitations should also be acknowledged. First, the cross‐sectional design precludes causal inference regarding the relationship between loneliness and nomophobia. Second, the use of self‐reported questionnaires introduces the possibility of reporting and social desirability biases. Third, participants were recruited from a single university, which may limit the generalizability of the findings to other educational settings and cultural contexts. Finally, loneliness explained only a small proportion of the variance in nomophobia, suggesting that additional psychological, behavioral, and environmental factors should be investigated in future research. Longitudinal and multicenter studies incorporating variables such as perceived social support, anxiety, stress, coping strategies, and patterns of smartphone use would provide a more comprehensive understanding of the determinants of nomophobia among university students.

## 5 Conclusion

Loneliness was identified as a statistically significant but weak predictor of nomophobia among Allied Medical Sciences students. Although loneliness independently contributed to nomophobia, it explained only a small proportion of the observed variance, suggesting that smartphone‐related anxiety is influenced by multiple psychosocial, behavioral, and academic factors. Interventions should therefore promote both psychological well‐being and healthy smartphone use, while future longitudinal multicenter studies should investigate additional determinants and causal pathways.

## Author Contributions

**Hasan Siamian:** conceptualization, investigation, writing – original draft, writing – review and editing, visualization, validation, methodology, project administration, formal analysis, software, resources, supervision, data curation. **Azita Balaghafari:** conceptualization, methodology, writing – original draft, writing – review and editing, resources. **Forouzan Elyasi:** conceptualization, methodology, resources, writing – review and editing, writing – original draft. **Shahram Mollania‐Jelodar:** conceptualization, methodology, formal analysis, data curation, writing – original draft. **Zahra Mahmoudvand:** conceptualization, methodology, resources, visualization, writing – original draft, writing – review and editing. **Azadeh Yazdanian:** conceptualization, writing – original draft, writing – review and editing, resources.

## Funding

The authors have nothing to report.

## Ethics Statement

This study was approved by the Ethics Committee of Mazandaran University of Medical Sciences (Approval No. IR.MAZUMS.REC.1401.10531) and was conducted in accordance with the principles of the Declaration of Helsinki.

## Conflicts of Interest

The authors declare no conflicts of interest.

## Transparency Statement

Hasan Siamian affirms that this manuscript is an honest, accurate, and transparent account of the study being reported; that no important aspects of the study have been omitted; and that any discrepancies from the study as planned have been explained.

## Data Availability

The data that support the findings of this study are available from the corresponding author upon reasonable request.
